# The complex of immunoglobulin A and uromodulin as a diagnostic marker for immunoglobulin A nephropathy

**DOI:** 10.1007/s10157-012-0617-3

**Published:** 2012-03-14

**Authors:** Takashi Obara, Sadaaki Mizoguchi, Yasunori Shimozuru, Toshitaka Sato, Osamu Hotta

**Affiliations:** 1 KAN Product Creation Unit, Eisai Product Creation Systems, Eisai Co., Ltd., 5-1-3 Tokodai, Tsukuba, Ibaraki 300-2635 Japan; 2 Hotta Osamu Clinic, 2-39 Rokutyonomeminamityo, Wakabayashi-ku, Sendai, Miyagi 984-0013 Japan

**Keywords:** IgA nephropathy, Diagnosis, Uromodulin, Immune complex, ELISA

## Abstract

**Background:**

The only tool to diagnose immunoglobulinn A nephropathy (IgAN) is renal biopsy which requires hospitalization; moreover, renal biopsy has a risk of critical bleeding. Therefore, a non-invasive method for accurate diagnosis of IgAN is desirable and a must-to-have tool for the clinics. For this purpose, we evaluated the diagnostic value of the IgA–uromodulin complex in the urine of patients with IgAN for its feasibility and adequacy.

**Method:**

We determined the IgA–uromodulin complex as a candidate for a diagnostic marker of IgAN by immunoprecipitation, liquid chromatography−mass spectrometry (LC–MS) and Western blot analysis. The enzyme-linked immunosorbent assay (ELISA) for the IgA–uromodulin complex was developed and applied to urine samples obtained from various kidney disease patients.

**Result:**

One hundred and three of 126 urine samples (81.7%) from IgAN patients were positive for the IgA–uromodulin complex, while only 25 out of 94 urine samples (26.6%) in other kidney disease patients were positive. Sensitivity was 81.7%, specificity was 73.4%, and diagnosis efficiency was 78.2%. The complex was negative in eight urine samples obtained from patients with Alport syndrome which is almost impossible to discriminate from IgAN by routine urinalysis.

**Conclusion:**

Detection of the urinary IgA–uromodulin complex by ELISA is a useful non-invasive method to diagnose IgAN.

## Introduction

Immunoglobulin A nephropathy (IgAN) is the most common glomerulonephritis among primary glomerular diseases [1, 2]. It has a poor long-term prognosis, and the renal survival rates are presumed to be approximately 50–80% in a long-term follow-up of more than 10 or 20 years [3, 4]. Several treatment agents including angiotensin-converting-enzyme inhibitors [5], angiotensin II blockers [6], cAMP elevating agents [7], immunosuppressive agents [8], fish oils [9], and tonsillectomy have been reported to be effective in slowing the progression to end-stage renal failure. Moreover, recent studies from Japan indicated that tonsillectomy followed by treatment with steroids introduces clinical remission if treatment begins at the early stage [10–13]. Although the early detection of IgAN is very important not only to slow the progression but also to obtain clinical remission, the chance of early detection is limited because renal biopsy, which needs hospitalization and is associated with an unavoidable risk of critical bleeding, is the only tool to give a definite diagnosis of IgAN. Therefore, a non-invasive method for accurate diagnosis of IgAN is desirable and a must-to-have tool for the clinics.

In this context, several candidates in urine such as the IgA–fibronectin complex [14], and proteomics [15] have been proposed. Recently, urinary uromodulin fragment was reported as a candidate marker by use of matrix-associated laser desorption/ionization-time of flight mass spectrometry [16]. To our knowledge, however, no practical marker with sufficient specificity and sensitivity has been developed to date.

Urinary IgA and IgA–IgG complex levels are high in IgAN patients [17]. In this study we examined the urinary IgA immune complex (IC) and determined proteins that combine with IgA. We then evaluated the diagnostic value of the urinary IgA–uromodulin complex by ELISA and showed that the IgA–uromodulin complex could be a good clinical diagnostic marker of IgAN.

## Method

### Patients and urine samples

In the first study (ELISA result of disease urine samples—a widely used test among kidney diseases), urine samples were obtained from various forms of biopsy-proven kidney disease patients including IgAN (95 patients), membranous nephropathy (MN; 18 patients), lupus nephritis (SLE; 5 patients), focal segmental glomerulosclerosis (FGS; 6 patients), minimal change nephrotic syndrome (MCNS; 3 patients), diabetic nephropathy (DMN; 5 patients), other kidney diseases (including amyloidosis, Alport syndrome, rapidly progressive glomerulonephritis, kidney sclerosis, kidney tumor, urethral lithiasis, etc.; 15 patients), and healthy controls (normal; 20 patients). Urinary protein and creatinine concentrations of each disease are shown in Table 1A.

**Table 1:** Concentration of urinary protein and creatinine

	Urine protein (mg/ml)	Urine creatinine (mg/dl)
(A) First study		
IgAN	0.55 ± 0.06	133.6 ± 7.8
MN	2.97 ± 0.68	121.4 ± 14.2
SLE	2.99 ± 0.133	116.0 ± 18.6
FGS	2.37 ± 1.05	112.7 ± 13.9
MCNS	5.03 ± 1.42	77.6 ± 33.5
DMN	2.31 ± 1.05	62.7 ± 19.8
Other kidney diseases	1.60 ± 0.46	106.8 ± 16.5
(B) Second study		
IgAN (before treatment)	0.75 ± 0.17	134.9 ± 11.8
Inactive IgAN (after treatment)	0.63 ± 0.13	96.8 ± 16.9
Alport syndrome	1.55 ± 0.45	82.9 ± 10.7
Amyloidosis	0.71 ± 0.20	78.4 ± 13.3
MPGN	1.32 ± 0.25	111.3 ± 41.3
ANCA-related nephritis	1.37 ± 1.11	50.8 ± 3.4
TBMD	0.23 ± 0.11	124.1 ± 50.0
FGS	2.68 ± 1.46	128.1 ± 39.6
Lupus nephritis (SLE)	2.45 ± 1.71	187.4 ± 116.0
DMN	1.36 ± 0.24	76.4 ± 34.7
MN	1.63 ± 0.33	94.1 ± 17.9
Hypertensive nephrosclerosis	0.25	30.8

In the second study (examination in other diseases groups—focused test to discriminate other diseases from IgAN), urine samples were obtained from various forms of biopsy-proven kidney disease patients exhibiting hematuria with or without proteinuria include IgAN (before treatment; 31 patients), and inactive IgAN; hematuria was no longer present after tonsillectomy with steroid pulse therapy (4 patients) [10–13], Alport syndrome (8 patients), amyloidosis (3 patients), membranoproliferative glomerulosclerosis (MPGN; 4 patients), anti-neutrophil cytoplasmic antibody (ANCA)-related nephritis (2 patients), thin basement membrane disease (TBMD; 2 patients), FGS (4 patients), SLE (2 patients), DMN (2 patients), MN (4 patients), and hypertensive nephrosclerosis (1 patient). Urinary protein and creatinine concentrations of each disease are shown in Table 1B.

### Immunoprecipitation (IP) method

Anti-human IgA antibody (Cappel Co.) was immobilized on Dynabeads^®^ M-450 Epoxy (Invitrogen Co.) according to manufacturer’s instruction and blocked with bovine serum albumin (BSA). A Tris–HCl buffered (pH 7.5) urine sample containing 0.15 M sodium chloride (NaCl) was mixed with anti-IgA-immobilized beads or control beads (BSA-blocked beads) and incubated overnight at 4°C. After washing with phosphate-buffered saline (PBS), proteins were eluted from beads with 0.1 M citric acid buffer (pH 3.0) and dialyzed against 1/10 concentration of PBS containing 0.01% sodium azide (NaN_3_), and concentrated.

### Identification of proteins combined with IgA in urine

Proteins recovered from the anti-IgA antibody affinity beads and control beads were separated by sodium dodecyl sulfate-polyacrylamide gel electrophoresis (SDS-PAGE). The proteins of interest were analyzed according to the method of Katayama et al. [18].

### Western blot analysis

The 3 μl of protein solution prepared by IP was separated by SDS-PAGE, and the proteins were then electrophoretically blotted onto a nitrocellulose filter (BA85; Schleicher & Schuell). After the filter was soaked in the blocking liquid [50 mM Tris–HCl (pH 7.5), 150 mM NaCl, 5% skimmed milk, 0.01% Tween 20, and 0.1% NaN_3_] at 4°C overnight, anti-human Tamm–Horsfall protein monoclonal antibody (Cedarlane Laboratories Ltd.) was added at 1/1000 dilution and incubated for 2 h at room temperature. After washing with the washing solution [50 mM Tris−HCl (pH 7.5), 150 mM NaCl, 0.01% Tween 20], HRP-conjugated anti-mouse IgG (Zymed Laboratories Inc.) was added to the washing solution at 1/1000 dilution and incubated for 1 h at room temperature and then washed with the washing solution. The membrane was developed by substrate solution [8.3 mM Tris–HCl (pH 6.5), 125 mM NaCl, 0.05% 4-chloro-1-naphthol, 0.01% hydrogen peroxide].

### Detection of a urinary IgA–uromodulin complex by ELISA assay

A ninety-six-well microtiter plate (NUNC, Polysorp) was coated with anti-human Tamm–Horsfall protein monoclonal antibody [10 μg/ml with 50 mM Tris−HCl (pH 7.5) and 0.15 M NaCl, 50 μl/well] at 4°C overnight. After washing three times with washing solution [50 mM Tris−HCl (pH 7.5), 150 mM NaCl, 0.01% Tween 20], wells of the plate were incubated with blocking solution [50% N102; Nippon-Yusi Co. Ltd., 25 mM Tris−HCl (pH 7.5), 75 mM NaCl, and 2% Block-Ace (Dainippon-Sumitomo Pharma Co. Ltd.)] at 4°C overnight and washed with the washing solution before use. Urine specimens diluted 1/50 with the dilution medium [50% N102; Nippon-Yusi Co. Ltd., 50 mM Tris−HCl (pH 7.5), 150 mM NaCl, and 2% Block-Ace (Dainippon-Sumitomo Pharma Co. Ltd.)] were added to the wells (50 μl each), and incubated for 1 h at room temperature. After washing three times with the washing solution, horseradish peroxidase (HRP)-conjugated goat anti-human IgA (Zymed) diluted with Can Get Signal^®^ Solution 2 (TOYOBO Co., Ltd.) at 1/3000 dilution was injected into each well (50 μl/well), and left to react for 1 h at room temperature. After washing three times with washing solution, 3,3′5,5′-tetramethylbenzidine (TMB) Liquid Substrate System for ELISA (Sigma) (50 μl/well) was injected, and left to react for 30 min at room temperature. 0.5 M sulfuric acid was added (50 μl/well), and optical density (OD) was measured at 450 nm with wavelength correction at 650 nm.

## Results

### Comprehensive analysis of the IgA IC in urine

**Fig. 1:**
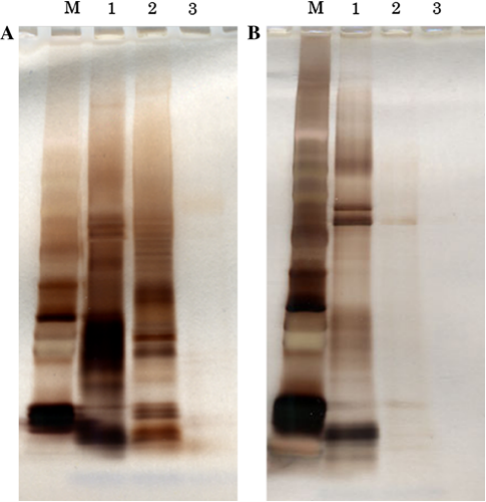
**a** PAGE of IP samples using anti-human IgA antibody-conjugated Dynabeads. ‘M’ represents the molecular weight markers. IP samples were derived from urine of IgAN patients (*lanes 1* and *2*) and a healthy control (*lane 3*). **b** PAGE of IP samples using BSA blocking Dynabeads. ‘M’ represents the molecular weight markers. IP samples were derived from urine of IgAN patients (*lanes 1* and *2*) and a healthy control (*lane 3*)

Proteins forming a complex with IgA in urine were isolated from two IgAN patients and a healthy control by using anti-human IgA antibody-immobilized beads and control beads. Isolated proteins were separated by SDS-PAGE (Fig. 1a). Compared with the urine of the healthy volunteer, many proteins were isolated from the urine of IgAN patients by IP using anti-human IgA antibody. In contrast, only a few proteins were identified from control beads (Fig. 1b). These results showed that proteins isolated from anti-IgA-immobilized beads specifically interacted with anti-human IgA antibody and many urine proteins exist as a complex with IgA in urine. To confirm the disease specificity of these protein complexes, IP experiments were performed using seven urine samples from patients with IgAN, and five samples from patients with other kidney diseases. Isolated proteins were analyzed and identified using LC–MS. Representative proteins are shown in Table 2.

**Table 2:** Summary of the LC–MS analysis result of the protein collected from the urine of IgAN patients and healthy donors by IP method using anti-IgA conjugated beads and BSA beads

Beads:			anti-IgA conjugated beads												BSA beads	
Disease:			IgAN							Other kidney diseases					IgAN	
Sample no:			1	2	3	4	10	11	12	5	6	7	8	9	1	2
	ID	Protein name														
Cell component or other	gi|340166	Uromodulin	3	3		1	3		1	1	1					
	gi68838	Aquaporin								1	1					
	gi|7331218	Keratin 1	2	2	2			1				2	1	2	1	2
	gi|34073	Cytokeratin 4 (408 AA)	1	1		1				1						
	gi186629	Keratin 10						1				1		1		
	gi|34033	Keratin 13	1	1												
	gi177139	Keratin 14				1		1					1	1		
	gi186685	Keratin 16						1	1				1			
	gi34081	Keratin 17										1				
Serum protein	gi|4557871	Transferrin	14	14			1						1		1	
	gi|28592	Serum albumin	3	45	6	2	4		3	2	1		5	3		3
	gi|4557385	Complement component 3 (C3)	1	3											1	
	gi|306882	Haptoglobin precursor	2	3												
	gi|72059	Leucine-rich alpha-2-glycoprotein	1	2											2	
	gi177827	Alpha-1-antitrypsin				1	2	2	2		1		2			
	gi45067732	S100 calcium-binding protein A9					1	2								
	gi|493852	Hemoglobin	5	1				1	1						8	2
	gi|224053	Macroglobulin alpha2	1	2												
Antibody component	gi|223099	IgA alpha1 Bur	2	1												
	gi|223335	Ig kappa L I Den	1	1												
	gi|229528	Protein Len, Bence-Jones	2	3											1	
	gi33700	Ig lambda light chain	1	2	1					1			1	1		
	gi9857759	IgG4 heavy chain											1			
	gi229526	Protein Rei, Bence-Jones			3								5			
		Ig kappa light chain	3	3									2			
		Ig heavy chain	2	4	2								1			

Urine samples were from IgAN patients (1, 2, 3, 4, 10, 11, 12), amyloidosis (5), SLE (6), DMN (7, 8), and MCNS (9). The numbers in the column show the identified number of fragments by LC–MS analysis

### Western blot analysis of the IgA–uromodulin complex

**Fig. 2:**
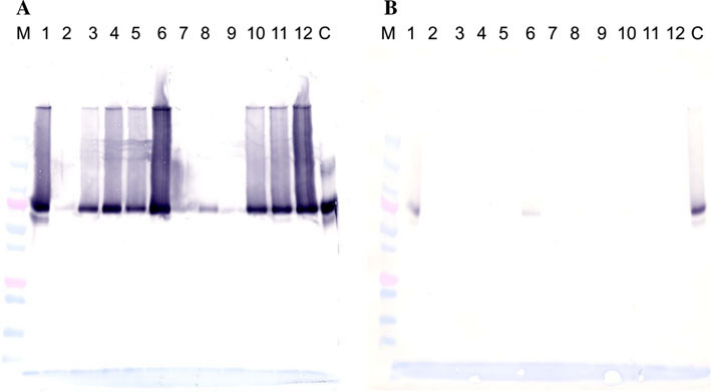
**a** WB analysis using anti-human uromodulin of IP samples using anti-human IgA antibody-conjugated Dynabeads. ‘M’ represents the molecular weight markers. ‘C’ represents control purified uromodulin. IP samples were derived from urine of IgAN patients (*lanes 1*, *2*, *3*, *4*, *10*, *11*, *12*), amyloidosis (*lane 5*), SLE (*lane 6*), DMN (*lane 7*, *8*) and MCNS (*lane 9*). **b** WB analysis using anti-human uromodulin of IP samples using BSA-blocking Dynabeads. ‘M’ represents the molecular weight markers. ‘C’ represents control purified uromodulin. IP samples were derived from urine of IgAN patients (*lanes 1*, *2*, *3*, *4*, *10*, *11*, *12*), amyloidosis (*lane 5*), SLE (*lane 6*), DMN (*lane 7*, *8*) and MCNS (*lane 9*). We can see only a weak band at *lane 2* in **a**; this seemed to be due to the loss of many beads because there was much fibrin precipitation in urine sample 2 in this experiment. A strong band was seen in the other experiment using urine sample 2 (data not shown)

The results of LC–MS analysis were confirmed by Western blot (WB) analysis using antibodies against the identified proteins. Figure 2 is an example of the analysis of uromodulin. Uromodulin was strongly positive in the urine samples of seven IgAN patients. In samples from patients with other kidney diseases, it was strongly positive in the urine of amyloidosis and SLE patients but very weak in other kidney diseases (Fig. 2a). Uromodulin was hardly detected in samples isolated by control beads (Fig. 2b). It was assumed that an IgA–uromodulin complex exists in the urine of IgAN patients and would be a diagnostic marker for IgAN.

### ELISA result of disease urine samples

**Fig. 3:**
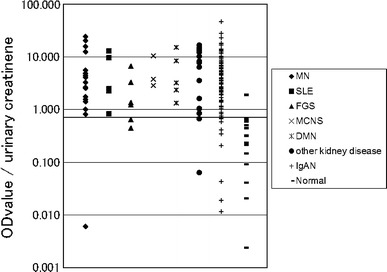
Distribution chart of measurements that detect the IgA–uromodulin complex in urine by ELISA. Cut-off line is drawn by ROC analysis in Fig. 4. We use 167 urine samples—18 MN, 5 SLE, 6 FGS, 3 MCNS, 5 DMN, 15 other kidney diseases, 95 IgAN, and 20 healthy controls (normal)

**Fig. 4:**
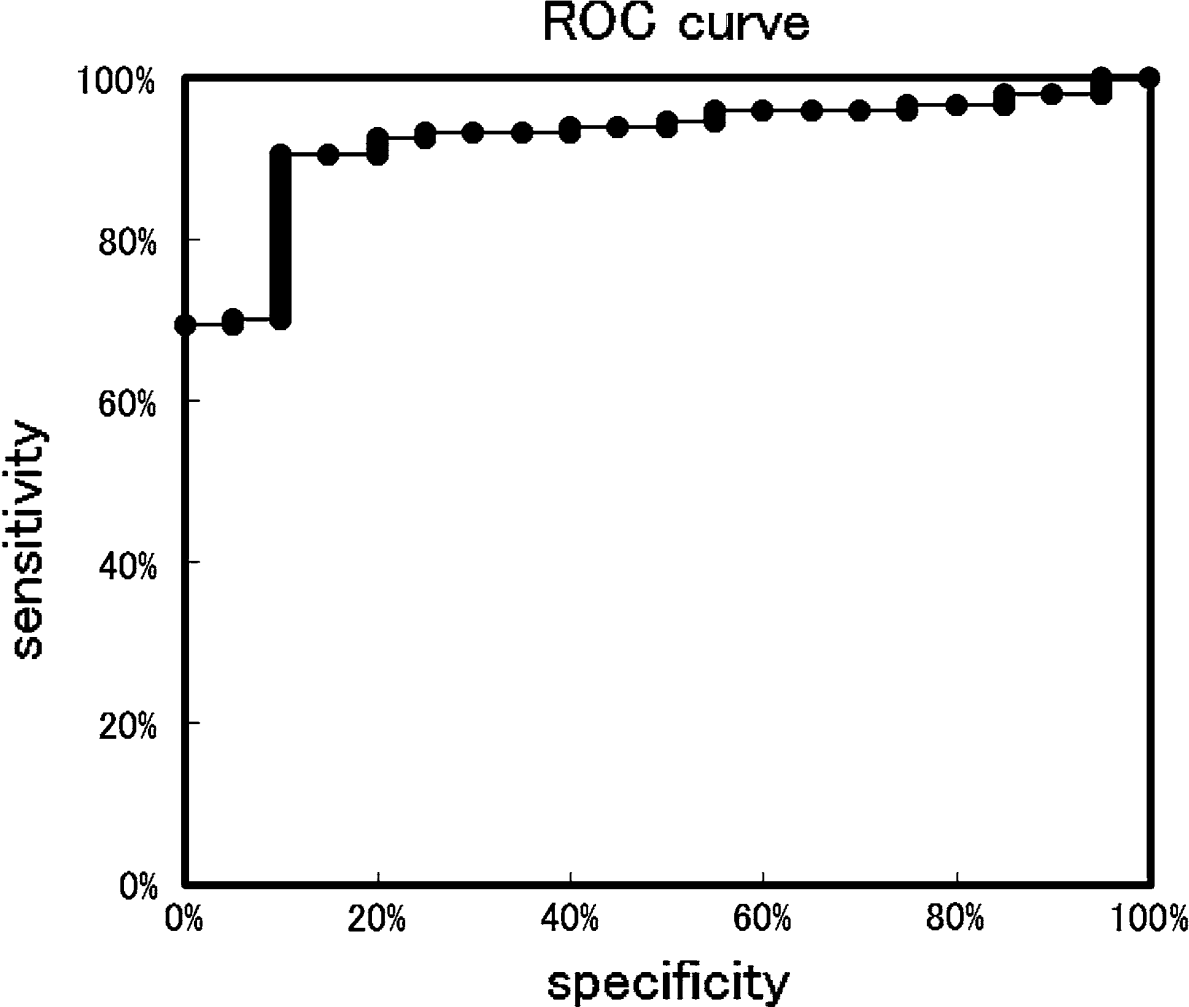
Result of the ROC analysis of measurements that detect the IgA–uromodulin complex in urine by ELISA in Fig. 3

The ELISA for the IgA–uromodulin complex was established using anti-human uromodulin antibody as the capture antibody and HRP-conjugated anti-human IgA antibody as the detection antibody. Figure 3 shows the results of the ELISA-tested 147 kidney disease samples, including 95 IgAN, and 20 healthy control samples. The OD values were adjusted for urinary creatinine concentration. Compared with healthy control samples, the magnitude of the IgA–uromodulin complex was significantly higher in IgAN samples, but no significant difference was found among other kidney diseases. Receiver operating characteristic (ROC) analysis was performed using the data from 147 kidney disease samples and 20 healthy control samples. The ROC curve is shown in Fig. 4. The cut-off value calculated from the ROC curve is 0.705, and the result of the positive rate of 147 kidney disease samples and 20 healthy control samples from the cut-off value is shown in Table 3. One hundred and thirty-three of 147 kidney disease patient samples were positive (90.5%) and only two samples were positive in 20 healthy controls (10.0%). Sensitivity was 90.5%, specificity was 90.0%, and diagnosis efficiency was 90.4%.

**Table 3:** Positive rate of kidney disease and healthy controls by ELISA for the IgA–uromodulin complex in Fig. 3

	Kidney disease patients	Normal
Total number	147	20
Positive number	133	2
Positive rate	90.5%	10.0%

Most of the patients were positive for proteinuria with a substantial amount of urine proteins; the IgA–uromodulin complex was found at various amounts, sometimes at high levels even though they were not diagnosed as IgAN (Table 1A). On the other hand, the ratio of the IgA–uromodulin complex compared to total urine protein was only high in cases of IgAN and not in other cases.

**Fig. 5:**
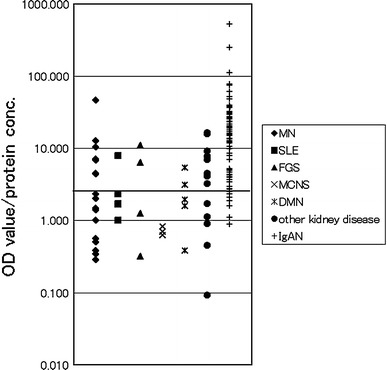
Distribution chart of the value of measurements that detect the IgA–uromodulin complex in urine by ELISA for each amount of urine protein. Cut-off line is drawn by ROC analysis in Fig. 6. 132 samples (133 ELISA-positive kidney disease samples except for one sample below the detection limit of pyrogallol red method) were analyzed. They included 17 MN, 5 SLE, 4 FGS, 3 MCNS, 5 DMN, 13 other kidney diseases and 85 IgAN

**Fig. 6:**
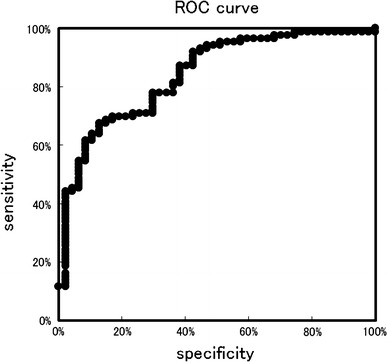
Result of the ROC analysis of the value of measurements that detect the IgA–uromodulin complex in urine by ELISA for each amount of urine protein in Fig. 5

In detail, the concentration of the urine protein of the specimen material that showed measurements higher than the cut-off value in urine was measured by the pyrogallol red method [19]. With the exception of one sample in which the concentration of the urine protein was below the detection limit, the amount of the IgA–uromodulin complex that had been obtained by the above-mentioned method was divided by the urine protein concentration, and the value of the complex for each urine protein amount was calculated. In other words, the concentration of the IgA–uromodulin complex adjusted for urinary creatinine was divided by a urine protein concentration adjusted for urinary creatinine; the results are shown in Figure 5. Samples from eighty-five IgAN patients and from 47 kidney disease patients (other than IgAN) were able to be clearly distinguished by comparing the value of the complex in the urine protein. Moreover, the ROC analysis of the samples from the 47 kidney disease patients (other than IgAN) and the samples from the 85 IgAN patients created the ROC curve shown in Fig. 6. The cut-off value calculated from the ROC curve was 2.45. The result of the positive rates of the 47 kidney disease patient samples (other than IgAN) and the 85 IgAN patient samples from the cut-off value is shown in Table 4. Seventy-nine samples of the 85 IgAN patient samples were positive (92.9%) and 20 samples of the 47 kidney disease patients were positive (42.6%) as shown in Table 4, and both were able to be distinguished clearly. Sensitivity at that time was 92.9%, specificity was 57.4%, and diagnosis efficiency was 80.3%.

**Table 4:** Positive rate of IgAN and other kidney diseases by ELISA for the IgA–uromodulin complex for each amount of urine protein in Fig. 5

	IgAN	Other kidney diseases
Total number	85	47
Positive number	79	20
Positive rate	92.9%	42.6%

### Examination in other diseases groups

**Fig. 7:**
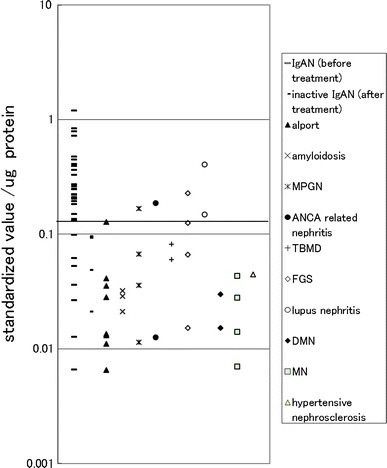
Distribution chart of the value of measurements that detect the IgA–uromodulin complex in urine in ELISA for each amount of urine protein in other disease groups. A spindle was indicated as ratio to standard sample. Cut-off line is drawn by ROC analysis in Fig. 8. 67 samples were analyzed including 31 IgAN (before treatment), 4 inactive IgAN (after treatment), 8 Alport syndrome, 3 amyloidosis, 4 MPGN, 2 ANCA-related nephritis, 2 TBMD, 4 FGS, 2 lupus nephritis, 2 DMN, 4 MN, and 1 hypertensive nephrosclerosis

ELISA similar to the above-mentioned method was analyzed in another specimen material group which included 8 samples of Alport syndrome (that is difficult to distinguish clinically from IgAN) and four samples of inactive IgAN; hematuria is no longer present after tonsillectomy with steroid pulse therapy [10–13]. The amount of the complex detection obtained by the above-mentioned method divided in the density of the urine protein, and the value of the complex for each amount of the urine protein was calculated; the results are shown in Fig. 7. Thirty-one IgAN patient samples and 36 kidney disease patient samples (other than IgAN) were able to be distinguished clearly by comparing the value of the complex for each amount of urine protein.

**Fig. 8:**
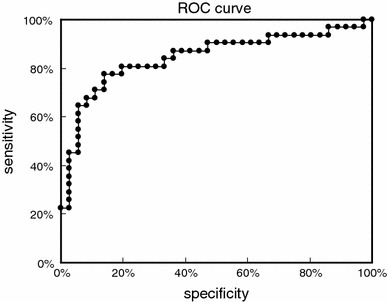
Result of the ROC analysis of the value of measurements that detect the IgA–uromodulin complex in urine by ELISA for each amount of urine protein on Fig. 7

Moreover, the ROC analysis of the samples from the 36 kidney disease patients (other than IgAN) and the 31 IgAN patients created the ROC curve shown in Fig. 8. The cut-off value calculated from the ROC curve was 0.130. Twenty-four samples from 31 IgAN patients were positive (77.4%) and 5 samples from 36 kidney disease patients (other than IgAN) were positive (13.9%) as shown in Table 5, and both were able to be distinguished clearly. Sensitivity at that time was 77.4%, specificity was 86.1%, and diagnosis efficiency was 82.1%. When the IgA–uromodulin negative samples were included, the sensitivity was 75.0% (24/32), the specificity degree was 88.1% (37/42), and the diagnosis efficiency was 82.4% (61/74).

**Table 5:** Positive rate of IgAN and other kidney diseases by ELISA for the IgA–uromodulin complex for each amount of urine protein in Fig. 7

	IgAN before treatment	Other kidney diseases
Total number	31	36
Positive number	24	5
Positive rate	77.4%	13.9%

In particular, four samples of inactive IgAN were judged to be negative and all eight samples of Alport syndrome, which is difficult to discriminate with IgAN by urinalysis, were judged to negative. These facts show this urinary marker to be very effective in a clinical diagnosis.

## Discussion

In this study, it was clarified that IgAN can be identified with a diagnosis rate of approximately 80% by measuring the complex of uromodulin and IgA in urine, and calculating the density per amount of urine protein. Uromodulin, also known as Tamm–Horsfall glycoprotein, is a glycophosphatidyl inositol (GPI)-anchored glycoprotein and is the most abundant protein in normal urine. It is produced by the thick ascending limb of the loop of Henle in mammalian kidneys. While the monomeric molecule has a molecular weight of approximately 68 kDa, it is physiologically present in urine in large aggregates of up to several million daltons [20]. Uromodulin may act as a constitutive inhibitor of calcium crystallization in renal fluids [20]. Excretion of uromodulin in urine may provide defense against urinary tract infections caused by uropathogenic bacteria [21]. The amounts of uromodulin in the urine of the clinical specimens used in this examination were measured. The healthy controls and the kidney disease patients had similar concentrations of uromodulin in urine (data not shown). Although the possibility remains, urinary uromodulin may undergo minor constructional changes in IgAN as reported by Wu et al. [16]. Antibodies to Tamm–Horsfall protein have been seen in various forms of nephritis (e.g., Balkan nephropathy); however, it remains unclear whether there is any (patho-) physiological relevance to these findings [22].

The level of urinary IgA and its complexes were reported to be higher in IgAN [17]. We have confirmed the level of urinary IgA is higher in kidney disease than in healthy volunteers, but the value of IgA divided by urinary protein concentration is not much higher in IgAN than in other kidney diseases (data not shown).

We made new monoclonal antibodies which specifically recognize mesangial cells. The ICs of IgA and the unknown antigens recognized by these antibodies were also found in the urine of IgAN patients; however, these were not superior to the IgA–uromodulin complex in sensitivity (data not shown).

The urine of IgAN is known to have a rather high concentration of the albumin–uromodulin complex [23]. The IgA–uromodulin complex might include IgA–uromodulin–albumin complex, but this three-component complex is considered to be a minor component, because the concentration of the IgA−albumin complex was even lower than that of the IgA–uromodulin complex (data not shown).

Because the IgA–uromodulin complex is found in the urine of almost all kidney diseases by ELISA, it does not seem to be specific to IgAN. Many kinds of proteins are found from IgA complexes that exist in the urine of patients with IgAN (Fig. 1a); IgA itself might tend to bind to some kind of proteins. Underglycosylated IgA which is found in IgA of IgAN patients seems to be adherent to some proteins, such as IgA, fibronectin, etc. [14]. Uromodulin seems to be a protein of this kind. The IgA–uromodulin complex might be a marker of IgAN in a similar way as HbA1c in diabetes; however, the mechanism and the meaning where such a complex is formed are problems that are still uncertain, and needs to be clarified in the future.

Our results indicated that IgAN can be discriminated from other proteinuric kidney diseases such as DMN, MN, FGS and MCNS by the value of the urinary IgA–uromodulin complex. In addition, it is advantageous in a clinical diagnosis to be able to distinguish IgAN from other hematuria-positive glomerular diseases such as Alport syndrome and TBMD. Henoch–Schönlein disease is another disease in this category, but unfortunately we were not able to obtain specimens from these patients in this study. On the other hand, however, it was relatively difficult to discriminate between lupus nephritis and IgAN by only using the value of the IgA–uromodulin complex; this was probably because of their similarity in terms of the histopathological development of the lesion, such as glomerular IgA deposits and glomerular vasculitis. However, IgAN can be easily discriminated from lupus nephritis based on serological examination such as anti-nuclear antibody, anti-DNA antibody and compliment levels. Thus, the difficulty of discriminating between IgAN and lupus nephritis by our method does not seem to be a crucial disadvantage for clinicians.

As mentioned earlier, the value of the IgA–uromodulin complex tends to be higher not in inactive IgAN having no hematuria but in the earlier phase of the disease in which inflammatory activity is still active. This could be an advantage because the combined treatment with tonsillectomy and glucocorticoid pulse therapy which can potentially prevent patients from end-stage renal failure is only effective if the intervention can be conducted in the early stage of the disease. In this sense, the value of IgA–uromodulin should be helpful for the selection of appropriate patients for whom this type of combined therapy could be beneficial [10–13].

It is needless to say that non-invasive measurement is more desirable than invasive in order to reach an exact diagnosis or selection of the therapeutic measurement. In fact, hesitation in performing renal biopsy often causes a delay in diagnosis and initiation of treatment in managing patients having asymptomatic hematuria and proteinuria.

The IgA–uromodulin complex, especially compared to total urine protein, could effectively detect IgAN by differentiating it from other glomerular diseases. Its value is also supportive in selecting appropriate patients for whom the combined tonsillectomy and glucocorticoid pulse therapy is likely to be effective to avoid further deterioration of IgAN pathology. Although renal biopsy may be unavoidable to reach a definite diagnosis, it should be still worthwhile to test the IgA–uromodulin complex prior to these techniques because of its benefits and easy-to-conduct nature.

IgAN is one of the most frequent causes of end-stage renal diseases. Furthermore, the beginning of IgAN is subjectively asymptomatic but only symptomatic in the urinalysis. Moreover, as early treatment intervention is necessary to obtain clinical remission [24], detection of IgAN in its early stage is very important. Thus, a non-invasive method of diagnosing IgAN by measuring the IgA–uromodulin complex would be of great benefit to a daily clinic to avoid the delay of initiating effective treatment. We believe it would drastically contribute to the improvement of current medical practice of renal diseases and ultimately provide great benefits to IgAN patients.
